# Effects of combining soil-applied insecticide and Bt corn for integrated pest management and resistance management of western corn rootworm (Coleoptera: Chrysomelidae)

**DOI:** 10.1093/jee/toae149

**Published:** 2024-07-10

**Authors:** John B McCulloch, Aaron J Gassmann

**Affiliations:** Department of Plant Pathology, Entomology and Microbiology, Iowa State University, Ames, IA, USA; Department of Plant Pathology, Entomology and Microbiology, Iowa State University, Ames, IA, USA

**Keywords:** *Bacillus thuringiensis*, economic threshold, pyramid–refuge strategy, resistance management, transgenic crop

## Abstract

The western corn rootworm, (*Diabrotica virgifera virgifera* LeConte, Coleoptera: Chrysomelidae), is a serious pest of corn (*Zea mays* Linnaeus, Cyperales: Poaceae) in the midwestern United States. Management practices for corn rootworm larvae include crop rotation, transgenic corn producing insecticidal toxins from the bacterium *Bacillus thuringiensis* Berliner (Bacillales: Bacillaceae) (Bt), and soil-applied insecticides. The extent to which combining soil-applied insecticide with Bt corn would be beneficial from the perspective of insect resistance management (IRM) or integrated pest management (IPM) remains uncertain. We conducted a 3-yr field study to characterize the implications of combining a soil-applied insecticide and Bt corn for IRM and IPM of western corn rootworm. Experimental treatments were Bt corn, a soil-applied insecticide, the combination of these factors, and an experimental control in which both factors were absent. Data were collected on root injury to corn by rootworm, survival to adulthood, adult size, and emergence time for western corn rootworm. We found that mortality caused by the soil-applied insecticide was insufficient to delay resistance to Bt corn. While combining Bt corn and a soil-applied insecticide may provide a short-term economic benefit, additional research is needed to determine appropriate economic thresholds for combining these tactics. Additionally, combining a soil-applied insecticide and Bt corn would not be sustainable over multiple growing seasons because of its potential to rapidly select for Bt resistance. In general, a more sustainable IRM strategy for rootworm management would include using crop rotation and alternating between non-Bt corn with soil-applied insecticide and Bt corn without soil-applied insecticide.

## Introduction

Western corn rootworm (*Diabrotica virgifera virgifera* LeConte, Coleoptera: Chrysomelidae) is a serious pest of corn (*Zea mays* Linnaeus, Cyperales: Poaceae) in the United States. It was first noticed as a pest in Colorado in 1909 (Gillette 1912, Spencer et al. 2005). Early reports suggest it was present as far west as Montana, and its range has since expanded east through Minnesota, Nebraska, and Ohio, including the corn-producing regions of the US Atlantic Coast, from the New England area to Georgia (Spencer et al. 2005, Gray et al. 2009, Meinke et al. 2009). The larvae, which feed on developing corn roots, are the most damaging life stage and can reduce yield by 15%–17% for every node of pruned roots (Dun et al. 2010, Tinsley et al. 2013). This pest has historically been managed using crop rotation and soil-applied insecticides targeting larvae or foliar insecticides targeting adults (Gillette 1912, Levine and Oloumi-Sadeghi 1991, Meinke et al. 2021). However, western corn rootworm has demonstrated its ability to evolve resistance to both crop rotation and insecticides (Levine and Oloumi-Sadeghi 1991, Levine et al. 2002, Gassmann et al. 2020, Meinke et al. 2021).

Transgenic crops producing insecticidal toxins from the bacterium *Bacillus thuringiensis* Berliner (Bacillales: Bacillaceae) (Bt) have been used to manage insect pests since 1996 (Tabashnik and Carrière 2019). Benefits of Bt crops include reduced use of insecticides, decreased pest injury to crops, and increased profits for farmers (National Academies of Sciences, Engineering, and Medicine [NASEM] 2016, International Service for the Acquisition of Agri-biotech Applications [ISAAA] 2019). In 2003, Bt corn targeting larval corn rootworm became available to farmers, and there are currently four Bt traits used for corn rootworm management (US Environmental Protection Agency [US EPA] 2023a). However, western corn rootworm has evolved resistance to all of these traits (Gassmann 2021).

The US EPA mandates an insect resistance management (IRM) plan for all Bt crops (US EPA 2023b). One IRM strategy is the refuge strategy, where non-Bt host plants are planted with the Bt crop. Refuges allow for the survival of susceptible individuals that can mate with resistant individuals surviving in the Bt portion of the field, thus keeping resistant alleles in a heterozygous state (Gould 1998, Andow 2008, US EPA 2023b). For single Bt traits, the inheritance of resistance is the key factor affecting the rate of resistance evolution, with resistance evolving slowest when resistance is recessive (Roush 1997, 1998; US EPA FIFRA Scientific Advisory Panel [US EPA FIFRA SAP] 1998). Non-Bt refuges can also be used with Bt crops that produce multiple toxins that target the same pest, which is defined as a Bt pyramid. Under the pyramid–refuge strategy, individuals resistant to one of the toxins in the pyramid are killed by the other toxin(s). A key factor affecting the rate of resistance evolution to a Bt pyramid is the level of mortality imposed on Bt-susceptible individuals by each toxin in the pyramid (Gould 1998, Roush 1998). For the pyramid–refuge strategy, refuges act to delay resistance by reducing the number of individuals that possess alleles for resistance to all Bt traits in the pyramid. Refuge plants can be planted interspersed within a field of Bt plants, by planting a mixture of Bt and non-Bt seed (i.e., refuge in the bag), or in blocks separate from but adjacent to the Bt crop (i.e., structured refuge). In the presence of refuges, pyramids are expected to delay resistance longer than sequential deployment of the same toxins if the mortality imposed by each of the individual toxins is at least 85% (Roush 1998).

Integrated pest management (IPM) provides a set of guiding principles for reducing yield losses to crops from insect pests (Pedigo et al. 2021). One of the tenets of IPM states management approaches should only be implemented if they increase profit. Pest abundance, economic losses caused by the pest population, commodity value, and management costs can all impact whether a management action will increase profit. Additionally, IPM approaches should focus on applying a diversity of tactics and consider the environmental impact of management strategies, in particular the use of insecticides. The goal of preventing a pest population from reaching an economically damaging size is based on the concept of an economic injury level, which is determined in part by the value of a commodity and the cost of management (Pedigo et al. 2021).

Populations of western corn rootworm will often grow and may exceed the economic injury level within a few generations if left unmanaged in continuous corn (Meinke et al. 2009). Prophylactic management with either insecticides or Bt traits is often practiced because of the need to make management decisions for larval rootworm when corn is planted (Meinke et al. 2009). Additionally, some farmers will add an at-planting application of soil-applied insecticide to Bt corn to reduce root injury if there is a perceived risk of resistance to Bt traits (Gray 2013). The ability to manage rootworm larvae with both Bt traits and insecticides raises the question of the merits of using soil-applied insecticides with Bt corn, and the extent to which this approach would be concordant with IPM and IRM approaches. In this study, we evaluated the effect of combining a soil-applied insecticide with Bt corn from the perspective of IRM and IPM. We evaluated the merits of this strategy to protect the root zone of corn plants and preserve yield. We also evaluated the IRM implications of such a pyramid to delay resistance to Bt corn, including mortality imposed by the soil-applied insecticide and the potential for the soil-applied insecticide to reduce the effectiveness of non-Bt refuges via effects on adult size, mass, and timing of emergence.

## Materials and Methods

### Experimental Design

This study was replicated in 3 years: 2018, 2019, and 2021. In each year, we used a randomized complete block design with 6 replicates at 2 locations. Both locations, Johnson Farm and Bruner Farm, are Iowa State University research and demonstration farms near Ames, in central Iowa. Past research found that the western corn rootworm populations at these locations are resistant to Cry3 proteins (i.e., Cry3Bb1, mCry3A, and eCry3.1Ab), with the fields used in this study corresponding to fields R1 and R2 in Gassmann et al. (2020).

This study utilized natural populations of corn rootworm that were drawn to the fields the previous year by a trap crop of late-planted corn with a variety of maturities. Each replication of this study contained 4 treatments: Bt corn with a soil-applied insecticide, Bt corn without the soil-applied insecticide, non-Bt corn with the soil-applied insecticide, and non-Bt corn without the soil-applied insecticide. The Bt hybrids used were G06Z97-5122, N75H-5122A, and G12W66-5222-EZ1 in 2018, 2019, and 2021, respectively, and the corresponding non-Bt hybrids were G06Z7-3120, N75H-GTA, and G12W66-Gt. In all years, the Bt hybrids produced rootworm-active Bt toxins mCry3A and eCry3.1Ab (events MIR604 and 5307; Syngenta Crop Protection AG, Basel, Switzerland) and the non-Bt hybrids were genetic isolines of the Bt hybrids. The soil-applied insecticide was Force 3G (active ingredient: tefluthrin, 3%, granular formulation), applied in-furrow at planting at the label rate of 465 mg/m (5 oz/1,000 feet) of row (Syngenta Crop Protection AG). Plots were planted between 28 April and 23 May, and fields were prepared using standard agricultural practices. Plots consisted of 4 rows, 6.1 m long, planted 0.76 m apart, with 15.2 cm between plants, equating to 86,487 plants per hectare (35,000 plants per acre). Heavy duty mesh cages with steel frames, 4 m long × 3.4 m wide × 2.4 m tall (Redwood Empire Awning, Santa Rosa, CA, USA), were erected over the plots to capture emerging adults. Cages were installed in mid-to-late June, before any adults had emerged. Each cage covered approximately 80 corn plants. Approximately 2 m of row with plants remained outside the cages. Corn plants had between 5 and 8 collared leaves (V5 to V8) when the cages were installed. In late June to early July, corn plants were trimmed to approximately 60 cm tall to facilitate collection of adult corn rootworm inside the cages.

Adult corn rootworm were collected from the cages on Mondays, Wednesdays, and Fridays. Adults were collected into 50 ml centrifuge tubes (Thermo Fisher Scientific, Waltham, MA, USA) using manual aspirators fitted into centrifuge tube caps (product #1135A, BioQuip, Rancho Dominguez, CA, USA). Collected adults were held in a freezer at −20 °C, prior to being counted. Species and sex were determined for each individual, with sex determined based on the morphology of the basitarsal pad following Hammack and French (2007). Root injury was evaluated by rating 5 plants per plot from the portion of the plot outside the cage. Roots were sampled in late July to early August and rated for larval rootworm feeding injury using the 0–3 node injury scale (Oleson et al. 2005). Field experiments were terminated in late September to early October in 2019 and 2021, after 3 consecutive collections yielded zero western corn rootworm adults at a location, or cumulative adult emergence had surpassed 99%. In 2018, the experiment was terminated on September 3 due to 3.8 inches of rain in a 9-day period, which prevented the continued collection of adults.

Head-capsule width and dry mass were determined for a randomly selected sample of 45 adults for each combination of treatment by sex by location by year (or all individuals were measured if there were less than 45 adults), which translates to 360 individuals per location per year and 2,160 individuals in total for all years of the study. Head-capsule width, defined as the widest distance at the eyes, was measured using a Leica MZ6 microscope (Leica, Wetzlar, Germany) with a digital camera and image analysis software (Moticam 10+ camera and Motic Images Plus 3.0(×64) software; Motic, Kowloon, Hong Kong, China). Software calibration was verified to the nearest 5 μm prior to every scoring session using a physical standard (KR-812 stage micrometer, Motic). After measuring head-capsule width, individuals were dried for 24–48 h at 60 °C (6503 Precision Oven, Thermo Fisher Scientific) and weighed to the nearest 0.001 mg with an analytical balance (model XS205, Mettler Toledo, Columbus, OH, USA).

### Data Analysis

Statistical analyses were performed using SAS 9.4 (SAS Institute, Cary, NC, USA). Data were transformed when appropriate to improve the normality of the residuals. Root injury data were square root transformed, and data for the average day of adult emergence for males and females in each plot were natural log transformed (Shrestha and Gassmann 2019). Dry mass of individual beetles was log_10_ transformed. Western corn rootworm emergence was standardized to adults per plant in each plot before being log_10_ transformed (Shrestha et al. 2016).

A mixed-model ANOVA was used (PROC MIXED) to test for the effects of corn type (Bt vs. non-Bt) and soil-applied insecticide (present vs. absent) on root injury and adult emergence. Fixed factors were corn type (Bt and non-Bt), insecticide (present and absent), and their interaction. We treated block as a random factor because of the random variation that arises among blocks which cannot be controlled or repeated (Sokal and Rohlf 1981). Random factors were block, which was nested within the interaction of year by location, and the interactions of all fixed factors with block nested within the interaction of year by location. The significance of each random effect was tested as the difference of the −2 RES log-likelihood in a model with, versus without, the random factor, with the resulting test statistic following a *χ*^2^ distribution with 1 df and a *P*-value based on a 1-tailed test (Littell et al. 1996). Plot was the independent experimental unit and the 5 corn plants dug per plot were treated as 5 subsamples per plot in the analysis of root injury. All pairwise comparisons for differences among means used a Tukey–Kramer adjustment.

Head-capsule width, dry mass, and timing of adult emergence were analyzed in a mixed-model ANOVA (PROC MIXED). Fixed factors were corn type (Bt vs. non-Bt), insecticide (present vs. absent), sex (male vs. female), and all interactions among these factors. Random factors were block, nested within the interaction of year and location, and all interactions of fixed factors with block nested within the interaction of year and location. Emergence time of adults was standardized by setting the first day of adult emergence at each farm and year combination to day 0 following Shrestha et al. (2016). All pairwise comparisons to test for differences between means used a Tukey–Kramer adjustment.

Mortality for each insecticidal treatment (Bt corn with insecticide, Bt corn without insecticide, and non-Bt corn with insecticide) was calculated in each block using the formula *Mortality = 100% × (1 − [Surv*_*trt*_*/Surv*_*control*_*])*, where *Surv*_*trt*_ = the number of adult western corn rootworm per plant in a treatment plot and *Surv*_*control*_ = the number of adult western corn rootworm per plant in the corresponding control plot of non-Bt corn without insecticide. PROC GLM was used to test for differences in mortality among the 3 insecticidal treatments, with a Tukey–Kramer adjustment for all pairwise comparisons. A 1-tailed *t*-test was used to test if the mortality of each insecticidal treatment was less than 85%, with a Bonferroni adjustment for 3 tests (PROC TTEST).

## Results

A total of 113,307 corn rootworm were collected in this study, consisting of 106,792 western corn rootworm, 4,386 northern corn rootworm, (*Diabrotica barberi* Smith and Lawrence, Coleoptera: Chrysomelidae), and 2,129 southern corn rootworm (*Diabrotica undecimpunctata howardi* Barber, Coleoptera; Chrysomelidae). Because northern corn rootworm accounted for <4% of the rootworm collected, data on this species were omitted. Additionally, southern corn rootworm were omitted because it is a subeconomic pest in the US Corn Belt and represented <2% of the rootworm collected (Szalinski and Owens 2003).

**Fig. 1.**
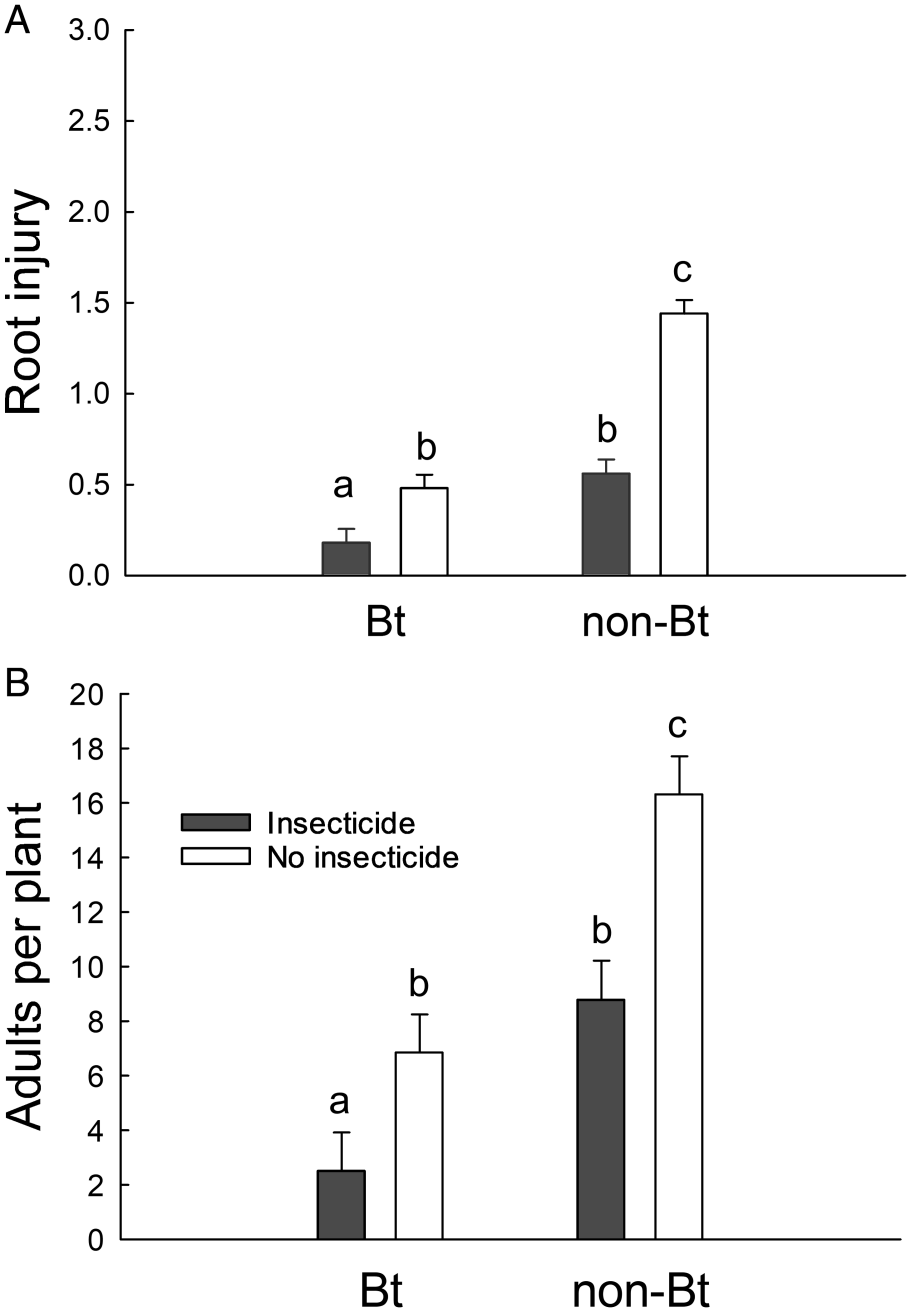
Insecticidal treatments reduce root injury and adult survival. A) Root injury (0–3 node injury scale from Oleson et al. (2005)) and B) western corn rootworm adults per plant for Bt and non-Bt corn, with and without the soil-applied insecticide. Bars are sample means, based on untransformed data, and error bars are the standard error of the mean. Root injury was square root transformed, and adult emergence per plant was log_10_ transformation, for use in a mixed-model ANOVA. Letters indicate significant differences between means after a Tukey–Kramer adjustment for all pairwise comparisons using the least-square means. Bt corn produced mCry3A and eCry3.1Ab, non-Bt corn was the non-Bt isoline of the Bt hybrid. Insecticide was tefluthrin (3% active ingredient, granular formulation) applied in-furrow at planting at a rate of 465 mg/m (5 oz/1,000 feet) of row.

The interaction of corn type and the soil-applied insecticide was significant for both root injury and survival to adulthood (Table 1). For both variables, pairwise comparisons revealed that all comparisons of were significantly different except between Bt corn without the soil-applied insecticide and non-Bt corn with the soil-applied insecticide (Fig. 1). The lowest root injury and adult emergence were observed in Bt corn with the soil-applied insecticide, and non-Bt corn without the soil-applied insecticide produced the highest root injury and adult emergence, while Bt corn without the soil-applied insecticide and non-Bt corn with the soil-applied insecticide had similar, and intermediate, values for these variables (Fig. 1).

**Table 1.** ANOVA table for root injury and survival to adulthood of western corn rootworm

Fixed effects				
	Root injury		Survival	
Source^a^	df	*F*-value	df	*F*-value
Corn	1,28	112.51**	1,29	130.97**
Insecticide	1,28	102.35**	1,29	77.71**
Corn × Insecticide	1,28	8.56*	1,29	19.24**

Random effects				
Source^a^	df	Root injury *χ*^2^		Survival *χ*^2^
Block (year × location)	1	10.10*		41.30**
Corn × block (year × location)	1	0.00		18.00**
Insecticide × block (year × location)	1	0.00		22.30**
Corn × Insecticide × block (year × location)	1	66.90**		0.00

^a^Bt corn produced mCry3A and eCry3.1Ab, non-Bt corn was the non-Bt isoline of the Bt hybrid. Insecticide was tefluthrin (3% active ingredient, granular formulation) applied in-furrow at planting at a rate of 465 mg/m (5 oz/1,000 feet) of row.*P < 0.01. **P < 0.0001. Root injury was square root transformed and survival per plant was log_10_ transformed for analysis.

**Fig. 2.**
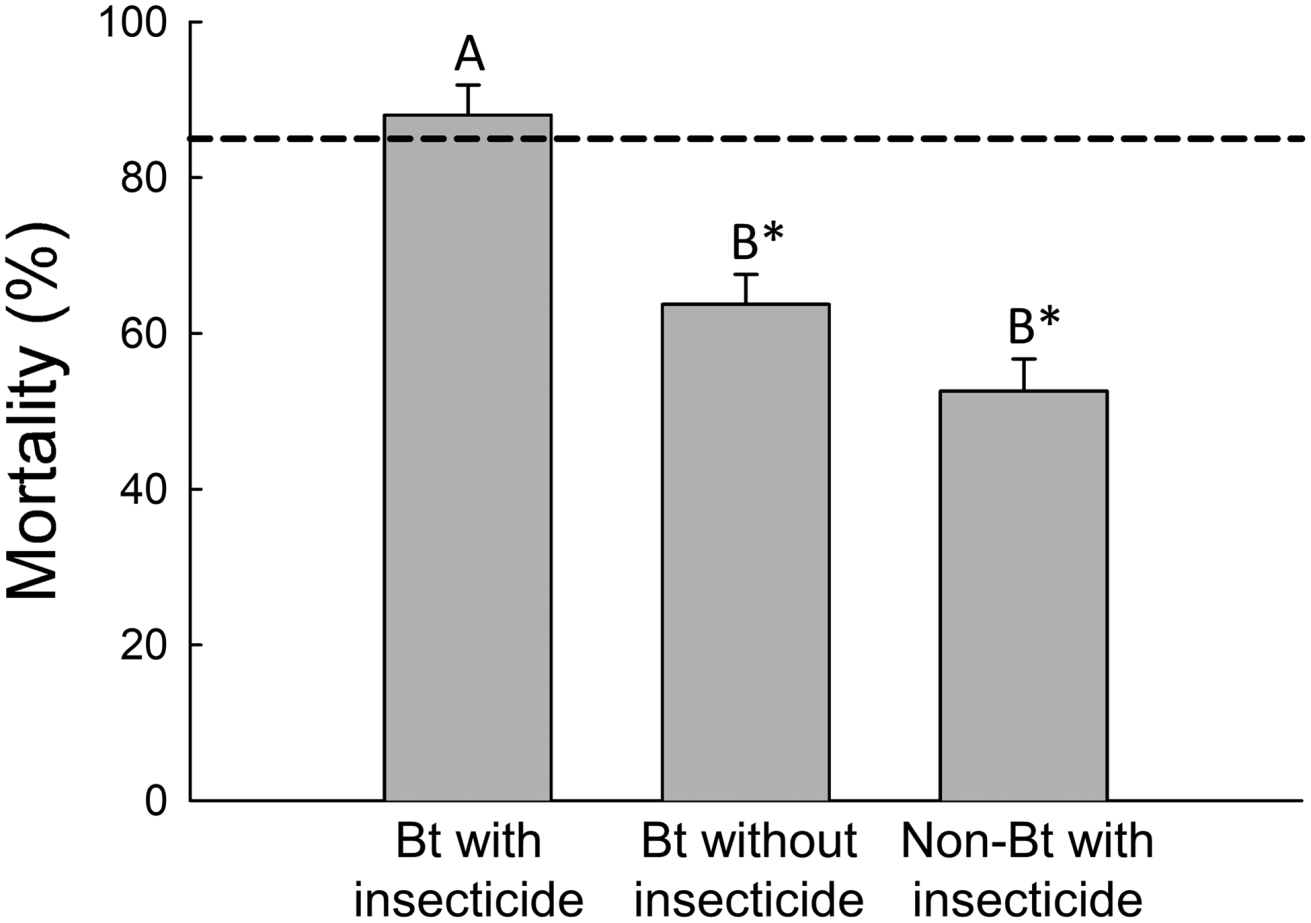
Mortality provided by each insecticidal treatment. Bars are mean mortality values and error bars are standard error of the means. The horizontal dashed line is 85% mortality, which is the threshold estimated in Roush (1998) for an insecticidal agent to delay resistance as part of a pyramid. Letters indicate significant differences between means after a Tukey–Kramer adjustment for all pairwise comparisons using least-squares means in a 1-way ANOVA. An asterisk indicates that the mortality of the treatment is significantly less than 85% in a 1-tailed *t*-test after a Bonferroni adjustment for 3 tests. Bt corn produced mCry3A and eCry3.1Ab, non-Bt corn was the non-Bt isoline of the Bt hybrid. Insecticide was tefluthrin (3% active ingredient, granular formulation) applied in-furrow at planting at a rate of 465 mg/m (5 oz/1,000 feet) of row. Percent mortality was calculated as *100% × (1 − [Surv*_*trt*_*/Surv*_*control*_*])*, where *Surv*_*trt*_ is the number of adults per plant in an insecticidal treatment and *Surv*_*control*_ is the number of adults per plant in the non-insecticidal control (i.e., non-Bt corn without insecticide). Survival in the control (i.e., non-Bt corn without insecticide) was used to calculate mortality for the insecticidal treatments and therefore mortality is only shown for insecticidal treatments.

The mortality caused by Bt corn with the soil-applied insecticide (88.07% ± 3.84) was significantly greater than either Bt corn without the soil-applied insecticide (63.80% ± 3.79) or non-Bt corn with the soil-applied insecticide (52.70% ± 4.02) (*F*-value = 21.51, df = 2,100, *P* < 0.0001) (Fig. 2). Mortality caused by Bt corn without the soil-applied insecticide and non-Bt corn with the soil-applied insecticide was not significantly different from each other (Fig. 2). Mortality was significantly less than 85%, the threshold for longer delays of resistance for a pyramid of insecticides compared to sequential use of 2 insecticides (Roush 1998), for Bt corn without the soil-applied insecticide (*t*-value = −5.73, df = 35, *P* < 0.0003) and non-Bt corn with the soil-applied insecticide (*t*-value = −5.80, df = 31, *P* < 0.0003). However, in the case of Bt corn with the soil-applied insecticide, the level of rootworm mortality did not differ from 85% (*t*-value = 1.76, df = 34, *P* = 2.868) (Fig. 2) (Roush 1998).

**Fig. 3.**
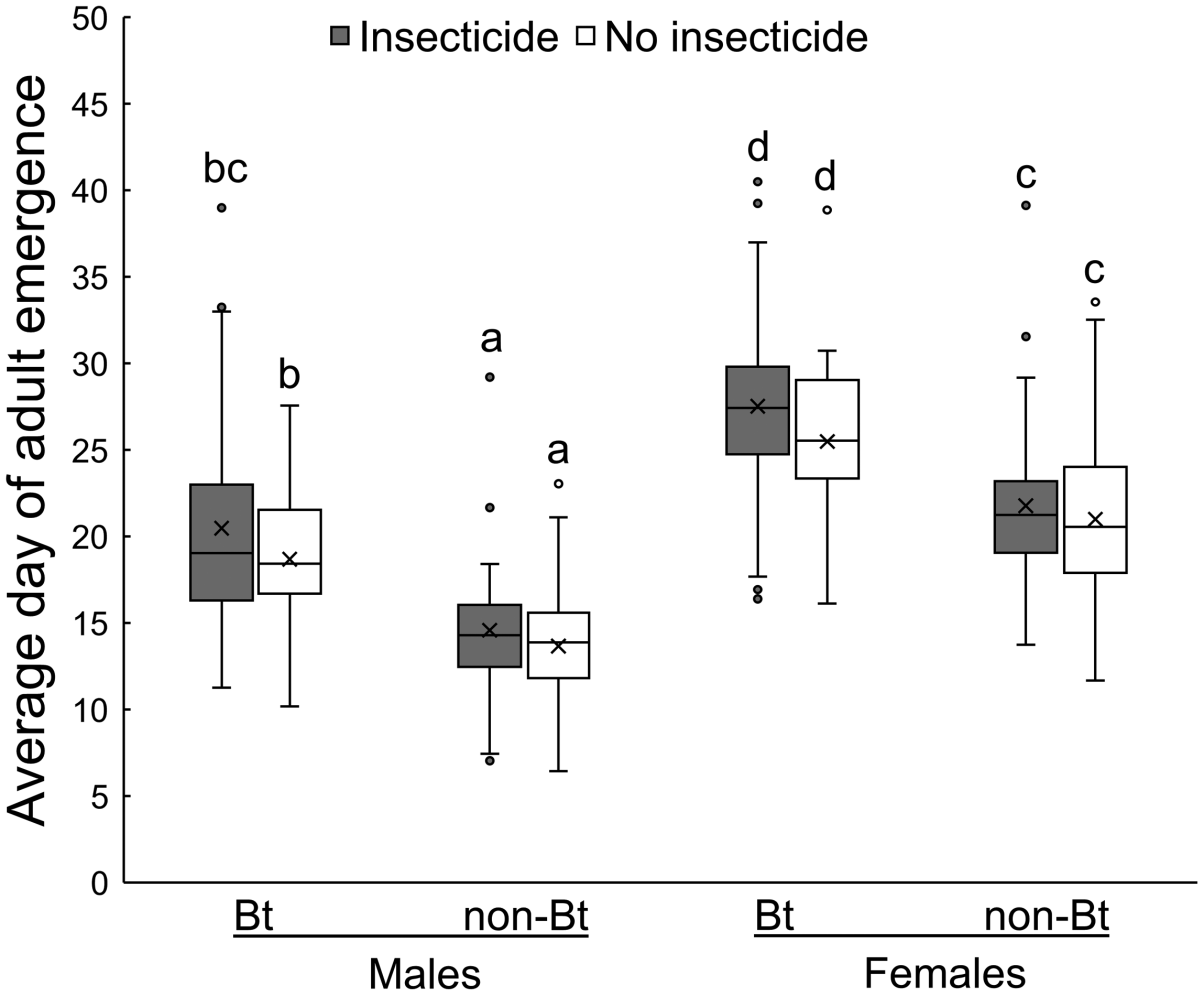
Differences in timing of adult emergence in Bt and non-Bt corn, with and without the insecticide. Untransformed average time to adult emergence for males and females from Bt and non-Bt corn with and without the soil-applied insecticide. Lines in the box indicate the median, X indicates the mean. The lower and upper bounds of the box are the 25th and 75th percentiles, respectively. The lower and upper ends of the whiskers are the 10th and 95th percentiles, respectively. Points outside the whiskers are outliers. Average time to emergence was natural log transformed for use in the analysis. Letters indicate significant differences between means after a Tukey–Kramer adjustment for all pairwise comparisons using least-squares means in a mixed-model ANOVA. Bt corn produced mCry3A and eCry3.1Ab, non-Bt corn was the non-Bt isoline of the Bt hybrid. Insecticide was tefluthrin (3% active ingredient, granular formulation) applied in-furrow at planting at a rate of 465 mg/m (5 oz/1,000 feet) of row.

The only significant factor affecting head-capsule width was sex, with females having a wider head-capsule width than males (females: 1133.29 ± 4.08 µm; males: 1125.79 ± 4.11 µm) (Tables 2 and 3). The soil-applied insecticide and sex significantly affected dry mass (Table 2). The soil-applied insecticide significantly increased adult mass, and females had greater mass than males (Table 3). Average timing of adult emergence was significantly affected by the interaction between corn type and sex, with the difference in emergence time on Bt corn versus non-Bt corn slightly greater for males (5.54 days) than females (5.24 days) (Table 2, Fig. 3). Emergence time for males on Bt corn and non-Bt corn was 19.71 ± 0.78 days and 14.17 ± 0.78 days, respectively, and emergence time for females on Bt corn and non-Bt corn was 26.71 ± 0.78 days and 21.48 ± 0.78 days, respectively. Additionally, emergence time was significantly later on corn with the soil-applied insecticide (21.33 ± 0.78 days) than on corn without the soil-applied insecticide (19.71 ± 0.78 days) (Table 2).

**Table 2.** ANOVA table for head-capsule width, dry mass, and average emergence timing of western corn rootworm

Fixed effects						
	HC^b^		Mass		Timing	
Source^a^	df	*F*-value	df	*F*-value	df	*F*-value
Corn	1,25	0.38	1,25	3.87	1,28	232.84***
Insecticide	1,25	2.47	1,25	10.03**	1,28	8.55**
Sex	1,25	5.72*	1,25	22.79***	1,28	722.53***
Corn × Insecticide	1,25	0.37	1,25	0.43	1,28	0.64
Corn × Sex	1,25	2.76	1,25	0.42	1,28	39.65***
Insecticide × Sex	1,25	0.00	1,25	0.88	1,28	0.07
Corn × Insecticide × Sex	1,25	1.80	1,25	0.89	1,28	0.29

	Random effects					
Source^a^	df	HC^b^*χ*^2^	Mass *χ*^2^		Timing *χ*^2^	
Block (year × location)	1	8.50**	10.40**		38.40***	
Corn × block (year × location)	1	0.10	1.80		0.00	
Insecticide × block (year × location)	1	0.00	1.10		2.70	
Sex × block (year × location)	1	0.20	1.10		3.80*	
Corn × Insecticide × block (year × location)	1	3.40	1.30		30.00***	
Corn × Sex × block (year × location)	1	0.00	0.00		0.00	
Insecticide × Sex × block (year × location)	1	0.00	0.00		0.60	
Corn × Insecticide × Sex × block (year × location)	1	0.00	0.00		0.00	

^a^Bt corn produced mCry3A and eCry3.1Ab, non-Bt corn was the non-Bt isoline of the Bt hybrid. Insecticide was tefluthrin (3% active ingredient, granular formulation) applied in-furrow at planting at a rate of 465 mg/m (5 oz/1,000 feet) of row. ^b^HC = head-capsule width. *P < 0.05. **P < 0.01. ***P < 0.0001. Mass was log_10_ transformed and timing was natural log transformed for analysis.

**Table 3.** Head-capsule width and dry mass for adult western corn rootworm in Bt and non-Bt corn with and without the soil-applied insecticide

Bt corn^a^	Insecticide^b^	Sex^c^	HC^d^ (µm)	Dry mass^d^ (mg)
n	n	f	1130 ± 4.5 (270)	1.92 ± 0.05^bc^ (270)
n	n	m	1122 ± 4.2 (270)	1.80 ± 0.03^ab^ (270)
n	y	f	1140 ± 4.1 (268)	2.03 ± 0.04^c^ (268)
n	y	m	1123 ± 3.7 (270)	1.80 ± 0.03^ab^ (270)
y	n	f	1132 ± 4.2 (268)	1.78 ± 0.04^ab^ (269)
y	n	m	1126 ± 4.1 (268)	1.65 ± 0.03^a^ (268)
y	y	f	1135 ± 4.1 (218)	1.94 ± 0.05^bc^ (218)
y	y	m	1138 ± 4.2 (210)	1.75 ± 0.04^ab^ (209)

^a^Bt corn produced mCry3A and eCry3.1Ab, non-Bt corn was the non-Bt isoline of the Bt hybrid. y = yes and n = no. ^b^Insecticide was tefluthrin (3% active ingredient, granular formulation) applied in-furrow at planting at a rate of 465 mg/m (5 oz/1,000 feet) of row. y = yes and n = no. ^c^f = female and m = male. ^d^The untransformed average is given with ±SEM, and the sample size is listed in parentheses. Dry mass was log_10_ transformed for use in the analysis. Letters indicate significant differences between means after a Tukey–Kramer adjustment for all pairwise comparisons using least-squares means in a mixed-model ANOVA. HC = head-capsule width.

## Discussion

In this study, the soil-applied insecticide did not impose a sufficient level of mortality against western corn rootworm larvae to function as a pyramid with Bt corn to delay evolution of Bt resistance (Fig. 2), a result that is consistent with past studies (Petzold-Maxwell et al. 2013, Shrestha et al. 2016). Effects on western corn rootworm survival, and on size and timing of adult emergence suggest that the soil-applied insecticide may not accelerate resistance evolution through effects on refuge insects when the rootworm population already possessed some level of Bt resistance. The significant reduction in root injury to Bt corn suggests that applying the soil-applied insecticide may be warranted in limited circumstances but additional research is needed to understand the appropriate economic thresholds in these cases (Fig. 1).

Reductions in root injury achieved by Bt corn will affect the extent to which the addition of a soil insecticide will further reduce root injury. When a western corn rootworm population is Bt-resistant and the Bt trait does little to reduce root injury, applying a soil-applied insecticide to non-Bt versus Bt corn achieves similar results (Shrestha et al. 2016). By contrast, when a western corn rootworm population is Bt-susceptible, and the Bt trait reduces root injury below the threshold for unexpected root injury, adding a soil-applied insecticide to Bt corn does not further reduce root injury (Petzold-Maxwell et al. 2013, US EPA 2013, Estes et al. 2016). The western corn rootworm populations in this study likely possessed some level of resistance to Bt corn, based both on past bioassays identifying Cry3 resistance in these fields, and the pervasiveness of Cry3 resistance in Iowa (Shrestha et al. 2018, Gassmann et al. 2020). Our data show when root injury to Bt corn was above the threshold for unexpected injury (0.5 nodes of injury for pyramid Bt corn), but significantly less than non-Bt corn, adding the soil-applied insecticide to Bt corn significantly reduced root injury compared with either tactic alone (Fig. 1A) (US EPA 2013). The addition of the soil-applied insecticide to Bt corn under these conditions also led to significantly greater reductions in emergence of adult western corn rootworm than was achieved by either Bt corn or the soil-applied insecticide alone, an effect which should aid in reducing western corn rootworm abundance (Figs. 1B and 2). Future research on the use of soil-applied insecticides to manage Bt-resistant rootworm on Bt corn should focus on the development of economic thresholds, as has been done for Bt-resistant bollworm (*Helicoverpa zea* (Boddie), Lepidoptera: Noctuidae), on Bt cotton (*Gossypium hirsutum* Linnaeus, Malvales: Malvaceae) (Del Pozo-Valdivia et al. 2021).

The potential role of combining a soil-applied insecticide with Bt corn to manage rootworm populations with incomplete Bt resistance is further supported by an economic analysis of the data from this study. We found that the addition of the soil-applied insecticide to Bt corn reduced root injury by 0.3 nodes (Fig. 1A). Past research has found that each node of pruned roots reduces yield by 15%–17% (Dun et al. 2010, Tinsley et al. 2013). The average yield for corn in Iowa for 2023 was 201 bushels per acre (12.6 metric tons per hectare) (United States Department of Agriculture – National Agriculture Statistical Service [USDA-NASS] 2024). As such, a 4.8% reduction in yield (16% × 0.3) would equate to approximately 9.6 bushels per acre (4.8% × 201). The average price of corn in 2023 was $6.05 per bushel (January–November), meaning that saving 9.6 bushels per acre would equal approximately $58 per acre (9.6 × $6.05) (USDA-NASS 2023). As long as the cost of insecticide per acre is less than the value of the yield protected, a farmer’s profit would increase if a soil-applied insecticide were used with Bt corn (Pedigo et al. 2021). With insecticide application costs potentially ranging from $16 to $26 per acre, pairing Bt corn with a soil-applied insecticide would increase profit in this situation (Bethlem 2023, Plastina 2023). However, this economic benefit is only expected to arise in situations where root injury to Bt corn is lower than that on non-Bt corn but still above the threshold for unexpected injury. Additionally, past research has found that continuous use of Bt corn can rapidly select for resistance, which would erode the additional protection, and profits, provided when using a soil-applied insecticide with Bt corn (Tabashnik and Carrière 2015, Dunbar et al. 2016, Gassmann 2021, Gassmann and Reisig 2023).

The proportion mortality caused by the soil-applied insecticide in our study was 52.70% (±4.02), significantly less than 85%, which is the threshold estimated by Roush (1998) for an insecticidal agent to delay resistance when placed in a pyramid (Fig. 2). This means that combining this soil-applied insecticide with Bt traits would not provide a resistance management benefit by delaying the evolution of Bt resistance (Roush 1998). However, more research would be beneficial to test the assumption that 85% mortality is the threshold below which mortality imposed by the individual toxins in a pyramid, including a pyramid of Bt corn with a soil-applied insecticide, would not delay resistance. Additionally, past research has shown that the field populations in this study were resistant to Cry3 traits, which may explain why the mortality imposed by Bt corn without the soil-applied insecticide was significantly less than 85% (Fig. 2) (Gassmann et al. 2020).

However, adding a soil-applied insecticide to Bt corn or to non-Bt refuges may act to accelerate resistance evolution if it decreases mating between Bt-susceptible individuals emerging from non-Bt refuges and Bt-resistant individuals emerging from Bt corn. If the addition of a soil-applied insecticide reduces adult emergence from Bt corn or the non-Bt refuge, the ability of a refuge to delay resistance could be increased or decreased, respectively. In a study examining Bt-susceptible populations of western corn rootworm, a soil-applied insecticide reduced adult emergence in non-Bt plots but did not further reduce adult emergence in Bt plots, indicating that a soil-applied insecticide would reduce the size of the refuge population and potentially increase the rate of Bt resistance evolution (Shrestha et al. 2016). Other studies have suggested that soil-applied insecticides are not useful for reducing populations of western corn rootworm because they do not protect the entirety of the root zone, allowing larvae to complete development on roots that have grown out of the protection zone of the insecticide (Gray et al. 1992, Estes et al. 2016). Our data show that the addition of the soil-applied insecticide reduced adult emergence in both Bt and non-Bt corn (Fig. 1B). However, because resistance was likely already prevalent in the populations evaluated in this study, it is likely that the response to selection would have been rapid, and that the continued planting of Bt corn, either with or without the soil-applied insecticide, would quickly erode the benefit of using Bt corn due to the continued evolution of resistance (Gassmann et al. 2011, Gassmann 2021, Gassmann and Reisig 2023).

Additional influences of soil-applied insecticides on the effectiveness of refuges to delay resistance evolution may arise from effects on adult size and the timing of adult emergence. The increased mass of adults emerging from plots treated with the soil-applied insecticide and the lack of size differences between adults emerging from Bt corn versus non-Bt corn suggests a lack of sublethal effects for these parameters, an effect that likely arose because of reduced overall survival in plots with the soil-applied insecticide and Bt corn (Tables 2 and 3). Other researchers have also reported a lack of size difference for western corn rootworm emerging from Cry3 corn, from both susceptible and resistant populations (Frank et al. 2011, Petzold-Maxwell et al. 2013, Shrestha et al. 2016). To the extent that either Bt corn or a soil-applied insecticide delay adult emergence, assortative mating may be promoted, which could reduce the effectiveness of refuges to delay resistance evolution (Gould 1998, Petzold-Maxwell et al. 2013). We observed a significant interaction between corn type and sex on the timing of adult emergence (Table 2). However, we suspect that the longer delay of 0.3 days, in males compared to females, may not be biologically relevant, as it takes 3–5 days for 55% of males to respond to female sex pheromone (Guss 1976). While both Bt corn and the soil-applied insecticide significantly affected the timing of adult emergence, we did not observe a significant interaction between corn type and the soil-applied insecticide, or a significant 3-way interaction of these factors with sex (Table 2). Consequently, the soil-applied insecticide did not appear to magnify the asynchrony in emergence between individuals from Bt and non-Bt corn, representing a situation such as an integrated refuge (i.e., refuge in a bag), where the soil-applied insecticide would be applied to Bt and non-Bt refuge plants alike (Table 2, Fig. 3). The soil-applied insecticide may increase the asynchrony of adult emergence from Bt corn versus non-Bt corn, and promote assortative mating, if it were only applied to the Bt portion of the field and not the non-Bt refuge, such as in a structured block refuge (Table 2, Fig. 3). Additionally, Onstad and Meinke (2010) found that insecticide use in a block refuge is expected to decrease the time to resistance to Bt traits. However, because resistance was likely already prevalent in the western corn rootworm populations studied here, the benefit of refuges in delaying resistance would be substantially diminished (Tabashnik et al. 2023).

Our data show that pyramiding a soil-applied insecticide with Bt corn can produce economic benefits in limited situations, where western corn rootworm populations may be partially, but not completely resistant to Bt corn traits. Despite the short-term economic benefits of pairing the soil-applied insecticide with Bt corn, the continuous use of Bt corn for western corn rootworm management increases the risk of rapid resistance evolution, and would erode the benefits of using Bt corn with the soil-applied insecticide (Petzold-Maxwell et al. 2013, Shrestha et al. 2016, Gassmann 2021). Additionally, an insufficient level of mortality imposed by this soil-applied insecticide does not support the IRM strategy of pyramiding Bt corn with this soil-applied insecticide to delay resistance to Bt corn, and similar results have been found for other soil-applied insecticides used for management of western corn rootworm (Petzold-Maxwell et al. 2013, Estes et al. 2016, Shrestha et al. 2016). Future research should address the possibility of an economic threshold for combining a soil-applied insecticide with Bt corn to manage Bt-resistant populations of western corn rootworm. However, because of the clear risk of rapid resistance evolution, such an approach should only be used as a short-term strategy. For long-term sustainable management of western corn rootworm, farmers should focus on crop rotation, or alternating between Bt corn without a soil-applied insecticide and non-Bt corn with a soil-applied insecticide in cases with limitations to crop rotation, and well-timed adult management to reduce population abundance when necessary (Gassmann 2012, 2021; Petzold-Maxwell et al. 2013; Martinez and Caprio 2016; Shrestha et al. 2016; Gassmann and Meinke 2024).
